# Cytogenetic and Genetic Abnormalities with Diagnostic Value in Myelodysplastic Syndromes (MDS): Focus on the Pre-Messenger RNA Splicing Process

**DOI:** 10.3390/diagnostics12071658

**Published:** 2022-07-07

**Authors:** Nathalie Douet-Guilbert, Benoît Soubise, Delphine G. Bernard, Marie-Bérengère Troadec

**Affiliations:** 1 Université de Brest, Inserm, EFS, UMR 1078, GGB, F-29200 Brest, France; benoit.soubise@univ-brest.fr (B.S.); delphine.bernard@univ-brest.fr (D.G.B.); 2 CHRU Brest, Service de Génétique, Laboratoire de Génétique Chromosomique, F-29200 Brest, France; 3 CHRU Brest, Centre de Ressources Biologiques, Site Cytogénétique, F-29200 Brest, France

**Keywords:** myelodysplasia, splicing, diagnosis, del(5q), chromosome abnormalities, SF3B1, LUC7L2, PRPF8, RBM22, SLU7, U2AF1

## Abstract

Myelodysplastic syndromes (MDS) are considered to be diseases associated with splicing defects. A large number of genes involved in the pre-messenger RNA splicing process are mutated in MDS. Deletion of 5q and 7q are of diagnostic value, and those chromosome regions bear the numbers of splicing genes potentially deleted in del(5q) and del(7q)/-7 MDS. In this review, we present the splicing genes already known or suspected to be implicated in MDS pathogenesis. First, we focus on the splicing genes located on chromosome 5 (*HNRNPA0*, *RBM27*, *RBM22*, *SLU7*, *DDX41*), chromosome 7 (*LUC7L2*), and on the *SF3B1* gene since both chromosome aberrations and the *SF3B1* mutation are the only genetic abnormalities in splicing genes with clear diagnostic values. Then, we present and discuss other splicing genes that are showing a prognostic interest (*SRSF2*, *U2AF1*, *ZRSR2*, *U2AF2*, and *PRPF8*). Finally, we discuss the haploinsufficiency of splicing genes, especially from chromosomes 5 and 7, the important amplifier process of splicing defects, and the cumulative and synergistic effect of splicing genes defects in the MDS pathogenesis. At the time, when many authors suggest including the sequencing of some splicing genes to improve the diagnosis and the prognosis of MDS, a better understanding of these cooperative defects is needed.

## 1. Introduction

Myelodysplastic syndromes (myelodysplasia or MDS) are clonal and acquired myeloid hematologic malignancies characterized by inefficient hematopoiesis. The risk of progression from MDS to acute myeloid leukemia (AML) varies from 15 to 30%, depending on the presence of chromosomal abnormalities and gene mutations. Indeed, MDS result mainly from the accumulation of somatic genetic abnormalities and, in particular, from cytogenetic aberrations in MDS that are clonal and recurrent. They are found in about 50% of de novo MDS and in more than 80% of secondary (therapy-related) MDS. They mostly represent unbalanced abnormalities with the loss of chromosomal material, and translocations are rare. Importantly, recurrent cytogenetic aberrations and their number (defining or not a complex karyotype) in MDS are of diagnostic value [1,2,3]. In cases of cytopenias, recurrent chromosome aberrations can be predictive of MDS even in the absence of morphological MDS characteristics [1]. However, so far, in the WHO reference classification of MDS, only one genetic aberration is characteristic of a specific MDS entity: it consists of the partial deletion of chromosome 5 (del(5q)). Deletion of chromosome 7 is also considered in the WHO MDS classification [2] since its presence precludes it from the ‘MDS with isolated del(5q)’ subtype. Additionally, those recurrent cytogenetic abnormalities are strong independent prognostic markers [4,5]. Chromosomes 5 and 7 bear important genes implicated in the splicing of pre-messenger RNA; those genes could be driver genes involved in the emergence or maintenance of MDS. Moreover, mutations in the gene, encoding the splicing factor SF3B1, present a strong diagnostic value when associated with ring sideroblasts. More rarely, MDS can emerge from a constitutive aberration: in particular, in the case of *DDX41* mutations, which is a gene that also encodes a splicing factor. Altogether, MDS can be considered ‘splicing diseases’. Highly recurrent mutations in genes, encoding splicing factors, are reported [6,7,8], with some having diagnostic or prognostic interests.

The goal of this review is to present the splicing genes that are already known, or suspected to be implicated, in the pathogenesis of MDS. First, we focus on the splicing genes located on chromosomes 5, 7, and on *SF3B1* gene. Indeed, del(5q), del(7q)/-7, and *SF3B1* mutations are the only genetic abnormalities in splicing genes with clear diagnostic values. Then, we present and discuss most of the other splicing genes that are showing a prognostic interest.

## 2. Chromosome 5 and Its Genes Implicated in Splicing

### 2.1. The Diagnostic Value of Chromosome 5 Deletion (del(5q) MDS)

In 1974, Van den Berghe et al. described the first recurrent chromosomal abnormality associated with MDS: the deletion of the long arm of chromosome 5 [9]. ‘MDS with isolated del(5q)’ was then included in the WHO reference MDS classification, as a distinct subtype, in 2001. In the last WHO 2016 MDS classification [2], the ‘MDS with isolated del(5q)’ is defined as the entity with: 1–3 dysplastic lineages, 1–2 cytopenias, none or any ring sideroblasts, less than 5% and 1% of blasts in the bone marrow and the peripheral blood, respectively, and importantly, with an isolated del(5q) or with one additional abnormality, except -7 or del(7q).

The deletion of 5q is the most frequent chromosomal abnormality reported in MDS (15%) [10]. It is of good prognosis when found alone or associated with another aberration (65% of the cases of del(5q)), and it is of poor prognosis when associated with two or more aberrations (complex karyotype) (33% of the cases of del(5q)) or in the presence of *TP53* mutations [3,4,10,11].

The 5q- syndrome is a distinct entity of MDS, with the sole 5q deletion and specific clinical and biological features (macrocytosis, anemia, normal or high platelet counts and hypolobulated megakaryocytes in the bone marrow, as well as peripheral and medullary blast counts inferior to 5%). Prognosis of patients with 5q- syndrome is excellent [12]. The 5q- syndrome is rare, according to Holtan et al. [13]. In 2022, with the evolution of classifications, the use of the term “5q- syndrome” is somehow confusing and potentially outdated.

**Figure 1:**
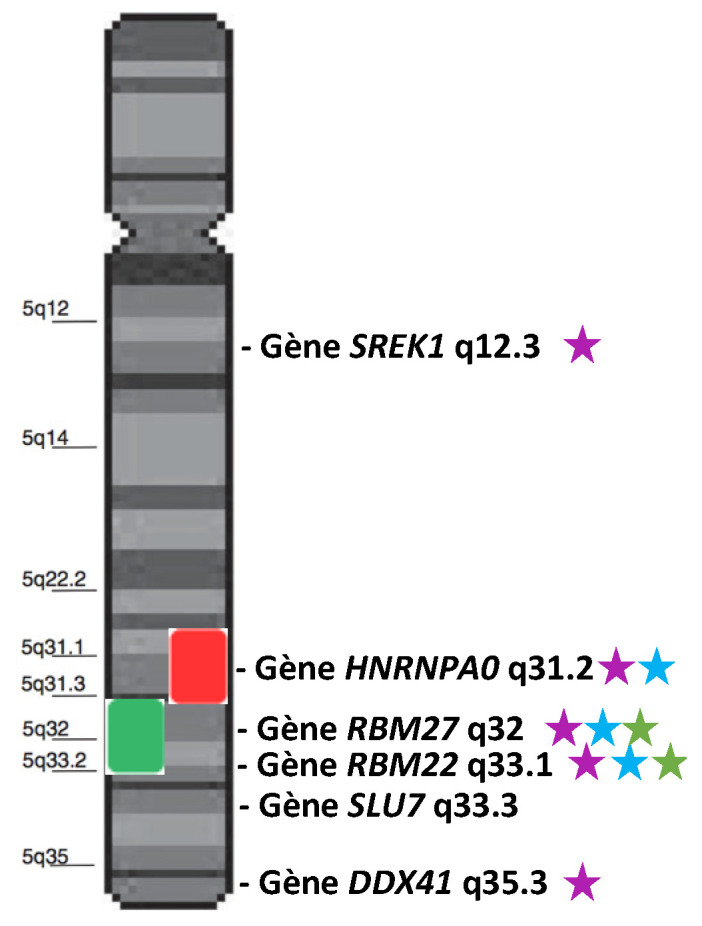
Schematic representation of chromosome 5 and two characteristic CDRs: the 5q- CDR (green box) and the higher-risk CDR (red box). Genes annotated as “splicing genes” are represented in front of their locus. Purple stars indicate the genes involved/mutated/dysregulated in MDS or hematopoiesis. Blue stars indicate the genes located in the most frequently deleted region. Green stars indicate the most dysregulated genes in the 5q- syndrome.

The borders of the commonly deleted region (CDR) of del(5q) slightly differ from one cohort to another [14,15,16,17]. Globally, it encompasses a large region from 5q14.1 to 5q35.1 [17]. Within this CDR, the 5q31.2–5q31.3 sub-region is more associated with higher-risk MDS, while the 5q32–5q33.2 sub-region is characteristic of 5q- syndrome. Several splicing genes are present in the 5q (Figure 1). We will focus on *HNRNPA0*, *RBM27*, *RBM22*, *SLU7*, and *DDX41* because some studies suggest/demonstrate their possible role in the cell cycle, proliferation, hematopoiesis, or in the pathogenesis of MDS.

### 2.2. Splicing Genes Located in the Commonly Deleted Region of the Deletion of 5q

#### 2.2.1. HNRNPA0 (Heterogeneous Nuclear Ribonucleoprotein A0)

*HNRNPA0* is located on chromosome 5, at the locus 5q31.2 in the human genome, and is expressed at reduced levels in CD34+ cells from patients with a del(5q) [18]. Its role is still poorly understood. This intronless gene encodes a minor heterogeneous nuclear ribonucleoprotein (hnRNP) bearing two RNA binding domains and a glycine-rich C-terminus [19]. It binds adenine and uracil (AU)-rich elements (ARE) of nuclear pre-messenger RNAs and stabilizes them. HNRNPA0 is involved in the control of the cell cycle, where it plays a role in DNA damage checkpoints, in particular through the post-transcriptional stabilization of p27(Kip1) and Gadd45α mRNAs [20]. HNRNPA0 is also implicated in the translation control system [21], in mRNA transport, and in nucleic acid metabolism [19].

HNRNPA0 is considered a tumor suppressor gene due to its overexpression in cancer. In humans, *HNRNPA0* is expressed in the hematopoietic tissue in lymphoid and myeloid lineages. In mice, depletion of *Hnrnpa0* alters the expression of myeloid specification genes and shifts the hematopoietic cell differentiation from monocytes to granulocytes [22]. In del(5q) MDS patients, different authors suggested a cooperative effect of *HNRNPA0* and *EGR1* double haploinsufficiency on the dysregulation of the myeloid differentiation [22,23]. Both genes, localized in the CDR of del(5q), are expressed at haploinsufficient levels in del(5q) MDS patients [18].

#### 2.2.2. RBM27 (RNA-Binding Motif 27)

*RNA-Binding Motif 27* (*RBM27*) is an RBM gene located on chromosome 5, at the locus 5q32. It is quite ubiquitously expressed in humans. *RBM27* encodes a protein containing an RNA-Binding Motif, which confers its ability to bind RNA. However, *RBM27* is still very poorly studied: in particular, in its role in pre-mRNA splicing. *RBM27* depletion by RNA interference causes an upregulation of a few alternative splicing events in the HepG2 cell line, such as tandem UTRs, exon skipping, and mutually exclusive exons [24]. The only described function of RBM27 is to be necessary for the Poly(A) Tail eXosome Targeting (PAXT) connection [25].

*RBM27*, which is located in the CDR of chromosome 5, is very frequently deleted and was identified as one of the most dysregulated genes in CD34+ cells of patients with the 5q- syndrome [14]. Nevertheless, its role in the pathogenesis of MDS remains unknown.

#### 2.2.3. RBM22 (RNA-Binding Motif 22)

**Figure 2:**
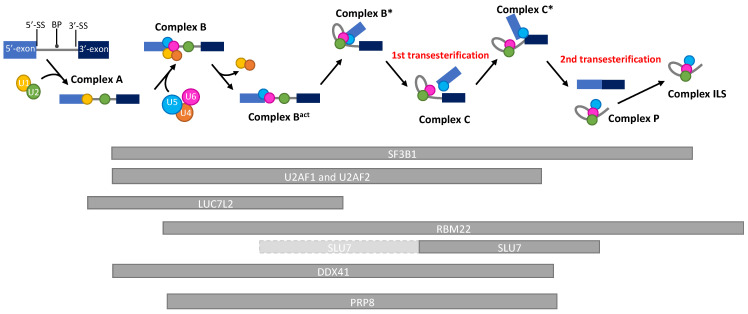
Schematic representation of the kinetics of the presence of splicing factors during a pre-mRNA splicing reaction. RNA splicing is a process by which introns are removed from pre-mRNAs, and exons are ligated to produce a mature mRNA. By the action of more than 200 proteins and 6 ribonucleoprotein complexes (U1-U6), the intron will be separated from the 5′-exon (1st transesterification) and, subsequently, from the 3′-exon (2nd transesterification), allowing the ligation of both exons. This reaction depends on the recognition of consensus splicing sequences (5′-Splice site, Branch point, 3′-Splice site) by the spliceosome. The 5′ and 3′ exons are represented with light and dark blue boxes, respectively. The U1, U2, U4, U5, and U6-snRNA are, respectively, represented with yellow, green, orange, blue, and pink circles. The kinetics of presence of the proteins discussed in this review are represented by grey bars below the reaction timeline. Light-grey bars with a dashed border line represent a low affinity presence. BP: Branch Point; 5′-SS: 5′-Splice site; 3′-SS: 3′-Splice site.

*RBM22* is located on chromosome 5 and at the locus 5q33.1 in humans. As *RBM27*, it is lost in almost all del(5q) MDS since it is localized in the del(5q) CDR (Figure 1). Belonging to the *RNA-Binding Motif* family of genes, *RBM22* encodes a protein containing an RNA-Recognition Motif (RRM) and a Zinc–Finger motif that allow it to bind RNA [26,27]. Its main role is to stabilize the catalytic core of the spliceosome (Figure 2). Indeed, RBM22 binds the RNA-maturing complex before the first transesterification (i.e., the separation of the intron from the 5′-exon, also called branching reaction) during the remodeling of the complex by Brr2 [28]. Mainly involved in the first step of splicing, RBM22 will tether several components together: contacting the ACAGA box and the Internal Stem Loop (ISL) of U6-snRNA, a few nucleotides of the intron downstream of the 5′-splice site and, most likely, as well as a few nucleotides of U2-snRNA [27,29,30]. It also interacts with other proteins, such as PRP19, Aquarius, and PRP8, which contribute to stabilizing the conformation of the catalytic core [29,31,32]. Of note; *PRPF8* (encoding PRP8) is also a gene mutated in MDS (see Section 5.2 below). Although its role is not clearly identified in the following steps of splicing, RBM22 stays in the complex during the whole process and leaves it after the intron release in the ILS complex [33,34]. Its presence in the spliceosome is essential, as its depletion leads to the failure of the branching reaction in yeast and several alternative splicing events in yeast, drosophila, or human cell lines [24,26,35]. Interestingly, RBM22 could potentially be involved in the calcium-dependent regulation of splicing, as it interacts with the calcium-binding protein ALG-2, and it is involved in the translocation of the latter and of another splicing factor (SLU7) from the cytoplasm to the nucleus and vice versa [36,37].

Furthermore, it was recently shown that RBM22 is also able to interact with DNA and act as a transcription factor, controlling the expression of about 3000 genes [38].

*RBM22* is a very conserved gene that is ubiquitously expressed in humans but slightly more in the bone marrow [39]. Though its exact role in hematopoiesis is still unclear, *RBM22* is undeniably involved in that process and linked to the occurrence of MDS. Indeed, located in the del(5q) commonly deleted region (CDR) of chromosome 5, *RBM22* is one of the most underexpressed genes in patients [14,18]. Its depletion induces significant defects in erythroid differentiation of CD34^+^ hematopoietic stem cells (HSC), which is one of the features of the 5q- syndrome and, thus, potentially links *RBM22* to the pathogenesis of this MDS entity [40]. Moreover, *Rbm22* was identified as an essential gene in mouse AML cell lines, and its in vitro downregulation was also associated with a defect of differentiation in the B cell lineage [41,42]. Together, those data suggest an implication of the loss of *RBM22* in del(5q) MDS pathogenesis [43].

### 2.3. Splicing Genes in the Variably Deleted Region of the 5q

#### 2.3.1. SLU7 (Synergistic Lethal with U5 snRNA 7)

*Synergistic lethal with U5 snRNA 7* (*SLU7*) is a gene located at the locus 5q33.3, and it has been identified, in 1992, in a screening of genes that could be synthetically lethal with U5-snRNA in yeast [44]. It encodes the splicing factor SLU7, which is involved in the selection of the 3′-splice site (3′-SS) for the second step of splicing (i.e., the nucleophilic attack of the 3′-SS by the 3′-OH of the 5′-exon, leading to exon ligation) (Figure 2). SLU7 arrives on the complex by low affinity binding on the intron 3′-tail (downstream of the branch point (BP)), during the B* step of splicing, before the first transesterification [45,46]. Then, this affinity becomes stronger before the second step of splicing, facilitated by the stabilization of SLU7 on the spliceosome by Prp22 [47]. For the second transesterification, SLU7 participates in the choice of the 3′-SS, forming a heterodimer with Prp18 [47]. It then facilitates the delocalization of Prp8, which was binding the 5′-SS and BP to make it bind both 5′-SS and 3′-SS, and it stabilizes this new conformation. Thus, SLU7 docks the 3′-SS into the catalytic center of the spliceosome, allowing the exon ligation to occur [45,47,48]. Interestingly, SLU7 is only necessary for the selection of 3′SS that are >7 nucleotides away from the branch point. Under that distance, the second step of splicing is able to occur properly without SLU7 [49]. When the distance is higher than 7 nucleotides, the splicing process stops before exon ligation [48]. Interestingly, the nuclear concentration of SLU7 can be modulated by translocating the protein to the cytoplasm. This process was shown under several stress conditions: calcium-stress, heat shock, or UV-C treatments. Further, the decrease in its nuclear concentration was associated with an effect on the alternative splicing of several genes [36,37,50].

Apart from pre-mRNA splicing, SLU7 is also involved in other biological processes, such as the cell cycle and genome stability. Indeed, the knock-down of *SLU7* in several human cell lines induces the formation of R-loops that cause DNA damages; it also affects mitosis by causing a defect in chromatid separation [51].

*SLU7* is rather ubiquitously expressed, with a sharp stronger expression in the brain, thyroid, testis, and bone marrow, as well as a lower expression in the pancreas, salivary glands, and liver [39]. So far, no link between *SLU7* and hematopoiesis has been made, and no correlation with hemopathies has ever been made. However, being located on the 5q chromosome, *SLU7* is susceptible to be deleted in del(5q) MDS, but it is not comprised of the so-called CDR (Table 1). Importantly, as already mentioned, RBM22 interacts with SLU7. Of note, apart from their interaction during the translocation of SLU7, they more importantly interact within the same complex of the spliceosome during the second step of splicing [52]. Thereby, *RBM22* happens to be co-deleted with *SLU7* in some cases. It would, thus, be interesting to question the effect of their co-deletion on the phenotype of del(5q) MDS.

#### 2.3.2. DDX41 (DEAD-Box Helicase 41)

*DDX41 (DEAD-Box Helicase 41)* is a gene located on the 5q35.3 cytogenetic band and belongs to DEAD box proteins with a putative RNA helicase function, and it seems to be a tumor suppressor gene. Involved in many cellular processes, such as splicing, rRNA processing, and innate immunity, DDX41 interacts with several spliceosome components, including SF3B proteins, PRP8 (*PRPF8* gene) scaffold proteins, as well as U2 and U5 complexes (Figure 2). Mutations of the *DDX41* gene disrupt the splicing progress and induce aberrant exon skippings or exon retentions [65].

Germline monoallelic (frameshifts) and somatic *DDX41* mutations in other alleles (located in DEAD box and helicase domain) are described in MDS and AML [66] (Table 1). First reported by Polprasert et al. in 2015 [54], these mutations are described as common mutations in adult MDS and AML, without known family history and with frequent preexisting cytopenia, normal karyotype, and favorable outcomes, according to Sebert et al. [67]. Moreover, Alkhateeb and colleagues reported a low incidence of *TP53* and splicing factor gene co-mutations in mutated *DDX41* patients [68].

*DDX41* is quite ubiquitously expressed. Low expression is described in 5q deletion [8]. Located on the 5q chromosome, *DDX41* is susceptible to being retained in del(5q) MDS and is rarely deleted in more complex karyotypes with five chromosome abnormalities. Some studies have described a responsiveness to lenalidomide in patients with *DDX41* mutations in myeloid neoplasms [69,70].

## 3. Chromosome 7 and Splicing Genes

### 3.1. Diagnostic and Prognostic Values

Deletion of 7q and monosomy 7 are among the most frequent chromosomal abnormalities reported in MDS (5%, respectively) [10]. They are mainly found in MDS-EB2, but can be retrieved also in the other MDS entities [11]. Deletion of 7q is associated with an intermediate MDS cytogenetic prognostic score. When associated with other chromosomal aberrations, monosomy 7 or del(7q) is of poor prognosis [4].

Several splicing genes are present in the chromosome 7, including *RBM28*, *RBM33*, *LSM5*, *TRA2A*, and *LUC7L2*. We will focus on *LUC7L2* because of the existing literature on the role of this gene in the evolution of MDS to AML.

### 3.2. LUC7L2 Gene

#### LUC7L2 (Lethal unless CBC 7-Like 2)

*LUC7L2* gene is located on chromosome 7 at the locus 7q34. *LUC7L2* encodes a pre-mRNA splicing factor bearing Zinc–Finger domains and a serine/arginine-rich (SR) domain in its C-terminal region, which is a domain that is often found in splicing factors. LUC7L2 protein is homologous to the yeast protein Luc7p—where it is essential for splicing—particularly for the recognition of non-consensus 5′ splice donor sites [71]. Two major spliced variants of *LUC7L2* can be expressed [72]. In mouse, Luc7l2 interacts with sodium channel modifier 1 (Scnm1) [72]. Yeast Luc7p is found in the early spliceosome pre-B complex, where it recognizes the duplex between the 5′ splice site and the 5′ end of U1 snRNA [73,74,75] (Figure 2). Similar results have been described in humans and mouse cells where LUC7L2 colocalizes with the U1 snRNP spliceosomal subunit: in particular, with U1 snRNP-specific protein U1-70K (SNRNP70) [71,72,73,76,77]. LUC7L2 preferentially binds to exonic sequences near, or at, the 5′SS, rather than to intronic sequences [76]. LUC7L2 either binds directly or not to exonic splicing enhancer (ESE) sequences (AAGAAG sequences) [76]. Alternative splicing events regulated by LUC7L2 have been recently published [57,76,77,78]. In particular, depletion of *LUC7L2* increases the expression of multiple spliceosomal factors that will result, directly or not, in the repression of oxidative phosphorylation and the promotion of glycolysis [76,77].

Loss of function of LUC7L2 alters the hematopoietic differentiation of induced pluripotent stem cells (iPSCs) [79].

In MDS, *LUC7L2* is located in the most commonly deleted region of -7/del7q MDS: 7q34. This region is deleted in 85% of -7/del7q MDS patients [80,81]. Loss of chromosome 7 in MDS has been associated with high-risk. The 7q deletion including *LUC7L2* and *EZH2* genes was also associated with mutations in *TP53*, *KRAS* and *IDH1* [80].

In 2012, a first report described a somatic heterozygous mutation (R27X) in *LUC7L2* in secondary AML [55] (Table 1). In 2013, the association between *LUC7L2* mutation (giving rise to the truncated LUC7L2 R279X variant) and the evolution from MDS to acute myeloid leukemia (AML) was reported [56]. *LUC7L2* mutations/deletions in MDS significantly affect the overall survival [7]. Mutations in *LUC7L2* gene are far less frequent than in *SF3B1*, *SRSF2*, and *U2AF1* genes [55,57,80]. Mutations in *LUC7L2* are described as hemizygous, heterozygous, and homozygous [57,81,82].

A lower expression of *LUC7L2* is reported in bone marrow samples of myeloid neoplasm patients (including MDS patients) compared to controls [57]. This low expression of *LUC7L2* dysregulates 5′SS selection and increases the splicing of normally retained introns (RIs) [57].

In particular, a 40% decreased expression of *LUC7L2* and other genes from the long arm of chromosome 7, compared to healthy controls., is reported in the CD34^+^ HSC from MDS patients, including some with monosomy 7 or del(7q) demonstrating the haploinsufficiency of this gene in MDS with monosomy 7 or del(7q) [83]. MDS samples with mutations in splicing factors other than *LUC7L2* (e.g., *SRSF2*) also harbor aberrant splicing of *LUC7L2* transcripts [57,84]. *LUC7L2* mutations in MDS -7/del7q patients have been associated with shorter overall survival relative to patients with normal *LUC7L2* expression [81].

## 4. The Only Gene with, So Far, a Diagnostic Value Is a Splicing Gene: *SF3B1*

### SF3B1 (Splicing Factor 3B Subunit 1)

The *SF3B1* gene, which is located on chromosome 2 (2q33.1), encodes an essential component of the U2 small nuclear ribonucleoprotein particle (snRNP) that is involved in the recognition of the branch point sequence close to the 3′ splice site during pre-mRNA splicing, thus participating in the definition of intron/exon junctions (Figure 2). SF3B1, which is the largest subunit of the heptameric protein complex Sf3b, contains a highly conserved HEAT domain composed of 20 tandem repeats structured as a superhelix, in C-terminus. SF3B1 interacts with other proteins outside the U2 complex, such as Histone proteins [85] or DNA-repair proteins (FANCI, FANCD2) [86], suggesting U2-independent functions of SF3B1. *SF3B1* is a ubiquitous gene, found to be essential for hematopoiesis in zebrafish models [87]. *SF3B1* is the most frequently mutated gene in MDS (15–30%) [7,59,60,62], especially in the group of MDS with ring sideroblasts (RS) for which approx. 80% of cases harbor an *SF3B1* somatic mutation [6,88,89]. Those are heterozygous missense gain-of-function mutations that affect amino acids at restricted sites in the H4 to H8 repeats of the HEAT domain [90,91]. K700E variant occurs in more than 50% of *SF3B*1-mutant MDS cases (Table 1). Importantly, cells mutated for splicing factors, including *SF3B1*, require the wild type allele for survival [92]. *SF3B1* mutations mainly alter 3′ splice site selection through the recognition of cryptic branch points, leading to hundreds of novel alternative/aberrant transcripts [93] whose contribution in the MDS pathogenesis is still poorly understood. More than 50% of these transcripts would be recognized and eliminated by the NonSense Mediated mRNA Decay mechanism [93], while others would lead to novel protein isoforms, as exemplified by a variant erythroferrone *ERFE* [94]. *SF3B1* mutations can also affect the splicing of its own pre-mRNA [95]. *SF3B1* mutations appear to occur early in the MDS pathogenesis, preceding other known genetic lesions [96,97]. Mutations in *SF3B1* are strongly associated with the presence of medullar ring sideroblasts (RS) [59], which represent erythroid precursors with abnormal iron accumulation in mitochondria. In the 2016 update of WHO classification, the criteria to define MDS with RS include the presence of *SF3B1* mutations, when as few as 5% of RS are at least detected [2]. Patients with *SF3B1* mutations showed significantly better overall survival and lower cumulative incidence of disease progression [89]. Recently, based on the analysis of a comprehensive data set of 3479 patients, the International Working Group for the Prognosis of MDS suggested to create an *SF3B1*-mutant MDS subtype as a distinct disease subtype, characterized by (i) cytopenia, (ii) somatic *SF3B1* mutation, (iii) isolated erythroid or multilineage dysplasia (for which RS will no longer be required for the diagnosis), (iv) bone marrow blasts <5% and peripheral blood blasts <1%, and (v) with selected concomitant genetic lesions as exclusion criteria [98]. This new *SF3B1*-mutant MDS entity has relatively good prognosis, and a potential response to luspatercept treatment. Nevertheless, all the *SF3B1* variants are unlikely to be equivalent. For instance, K666N was associated with an increased progression of MDS, as illustrated by an enrichment in high-risk MDS and AML, and a shorter overall survival compared to non-K666N *SF3B1*-mutant MDS cases [99]. Comparison of the clinical features of K700E and non-K700E *SF3B1*-mutant MDS patients shows that only K700E mutation independently predicts overall survival in MDS [100]. *SF3B1* mutations are mutually exclusive with *SRSF2* or *U2AF1* mutations in MDS, suggesting a synthetic lethality interaction, which has been confirmed in different models [92]. SF3B1 physically interacts with SUGP1, whose genetic alterations, which are rare in cancer, mimics *SF3B1*-mutant splice pattern [101]. Small molecules inhibitors against SF3B1 have demonstrated some effects on tumor cell death [102,103].

## 5. Other Mutated Splicing Genes with a Prognostic Value: *SRSF2, U2AF1, ZRSR2, U2AF2* and *PRPF8*

### 5.1. The Most Frequently Mutated Splicing Genes with a Prognostic Value: SRSF2, U2AF1, ZRSR2

#### 5.1.1. SRSF2 (Serine and Arginine Rich Splicing Factor 2)

The *SRSF2* gene, which is located on chromosome 17 (17q25.1), encodes an essential member of the serine- and arginine-rich (SR) protein family that plays an important role in constitutive and alternative splicing. SRSF2 promotes exon recognition by binding mRNA exonic splicing enhancer (ESE) motifs through its RNA recognition motif domain (RRM), located in the N-terminus. The SR domain facilitates the interaction between different SR splicing factors. *SRSF2* plays an essential role in hematopoiesis during embryonic development. *SRSF2* mutations occur in 20–30% of MDS [6]. Those are heterozygous missense mutations, mostly affecting P95 residue (P95H, P95L, P95R), located in an intervening sequence between the RRM and the SR domain (Table 1). Conditional expression of *SRSF2*^P95H^ impairs hematopoietic differentiation and promotes myelodysplasia in mice [61]. *SRSF2* mutations result in genome-wide alterations in ESE preference [61]. In contrast to *SRSF2* loss, *SRSF2* mutations alters the recognition of specific ESE motifs, leading to recurrent missplicing of key hematopoietic regulators, including the promotion of a poison exon of *EZH2* that undergoes NMD, resulting in reduced EZH2 protein level. Expression of hot-spot *SRSF2* mutation (P95H, P95R) in CD34+ cells leads to a dramatic inhibition of proliferation via a G2-M phase arrest and an induction of apoptosis [104]. Non-canonical functions of SRSF2, shared with other SR proteins, have been described in the mRNA life cycle, including the regulation of transcription elongation and the NMD mechanism [105,106]. SRSF2 plays also a role in the control of genomic instability [107], together with SF3B1 and U2AF1.

Mutations of *SRSF2* in MDS predicted shorter overall survival and more frequent AML progression compared with wild type *SRSF2* [60]. A meta-analysis performed on 10 cohort studies, covering 1864 de novo MDS patients, confirmed that *SRSF2* mutations had an adverse prognostic impact on overall survival and AML transformation [108]. Mutation status of *SRSF2* in patients with lower risk MDS was associated to shorter overall survival in several studies [108,109]. Importantly, targeting exon sequencing of 96 genes performed in 648 cytopenic patients, among which 212 were diagnosed with MDS, showed that variant alleles frequency for seven genes, including *SF3B1*, *U2AF1*, and *SRSF2*, could correctly re-classify subjects as either MDS or other in 74% of cases that were misclassified. This suggests that targeted sequencing of these genes could improve MDS diagnosis [110].

#### 5.1.2. U2AF1 (U2 Small Nuclear RNA Auxiliary Factor 1)

The gene U2AF1 (also known as U2AF35), which is located on chromosome 21 (21q22.3), encodes an essential accessory factor of U2 snRNP, which, together with U2AF2 (also known as U2AF65), binds to the AG dinucleotide at 3′ splice sites, thus ensuring an essential role in defining intron/exon junctions during the initial steps of pre-mRNA splicing (Figure 2). U2AF1 interacts with U2AF2 as a heterodimer, to form the U2 auxiliary factor (U2AF) complex. U2AF1 protein consists of two Zinc–Finger domains, as well as an UHM (U2AF homology motif) domain and an SR domain in C-terminal, which are both involved in the interaction with SRSF2. U2AF1 is required for survival and function of hematopoietic cells [111]. U2AF1 mutations occur in 11–16% of de novo MDS [6,63,112]. They are mainly heterozygous missense hot-spot mutations affecting amino acids S34 (S34F, S34Y) and Q157 (Q157R, Q157P) located in the N-terminal and C-terminal Zinc–Finger domains, respectively. Wild type allele of U2AF1 is required for cell survival [111]. RNAseq analysis performed in CD34+ hematopoietic cells expressing U2AF1^S34F^ (vs. wild type) showed exon-skipping events and alternative splice site usage in a restricted number of genes (affecting less than 1% of expressed junctions) [113], suggesting that mutant U2AF1 alter splicing in a context of specific RNA sequences. Importantly, mutations in SF3B1, SRSF2 and U2AF1 result in different splicing alterations, largely affecting different genes, but with an enrichment in RNA splicing and transport, protein synthesis, mitochondrial dysfunction and signaling cascades, suggesting common mechanisms of action in MDS [84,114]. U2AF1 hot-spot mutations were associated with inferior survival in several studies [62,115], but not in all studies [60]. VAF > 40% of U2AF1 was shown to be an independent factor of short overall survival in MDS patients, but the presence of co-mutated genes (such as ASXL1, RUNX1, TET2) with U2AF1 can affect disease progression and prognosis [63]. A meta-analysis covering 3038 patients from 13 studies showed that U2AF1 mutations were associated with poor survival in MDS patients, and patients with U2AF1^Q157^ had a worse OS than those with U2AF1^S34^ (Li et al., 2020). RNAseq analysis performed in myeloid malignancies *harboring U2AF1*^S34^ and *U2AF1*^Q157^ reveals that only 4% of the genes were commonly misspliced by both mutations [116], emphasizing the necessity to study more deeply the specific effect of each hot spot mutation and their respective incidence for prognosis in MDS.

#### 5.1.3. ZRSR2 (Zinc Finger CCCH-Type, RNA Binding Motif and Serine/Arginine Rich 2)

The gene *ZRSR2*, which is located on chromosome X (Xp22.2), encodes an essential component of the U12-dependent (minor) spliceosome [117,118], which excises a rare group of introns characterized by highly conserved 5′ splice sites and branch point sequences. More specifically, ZRSR2 is involved in the recognition of 3′SS during the early stages of spliceosomal assembly. Knockdown of *ZRSR2* in TF-1 cells leads to retention of U12-type introns, without affecting U2-type introns [117,118]. The protein ZRSR2, which shares structural similarities with U2AF35, consists in two Zinc–Finger domains flanking an UHM domain, with an SR domain at C-terminus. ZRSR2 physically interacts with U2AF2, as well as with SRSF1 and SRSF2 [119]. *ZRSR2* somatic mutations, which occur in approx. 3–7% of MDS [6,7,60] are widely distributed along the entire coding region, and correspond to nonsense or frameshift changes or alteration of splicing donor/acceptor sites, consistent with loss of function. ZRSR2 mutations impair splicing of U12-type introns by the minor-spliceosome pathway, mainly through increased intron retention [117,118], affecting several crucial genes involved in cell cycle, signaling, RNA binding and transport. Induced deletion of *Zrsr2* in mice revealed enhanced self-renewal of *Zrsr2*-deficient male and female hematopoietic cells, in contrast to the effects of hot spot mutations in *Sf3b1* or *Srsf2* [120]. In MDS *ZRSR2*-mutant samples, 48% of minor introns exhibited significantly increased retention [120]. In particular, minor intron retention in *LZTR1* (a cullin-3 adaptor for ubiquitin-mediated suppression of RAS-related GTPases), which correlated with reduced LZTR1 protein in MDS patients, resulted in cytokine independence. Patients with *ZRSR2* mutations were not found to have a higher transformation rate to AML [60]. Prognostic impact of *ZRSR2* mutations in MDS is currently unknown.

### 5.2. Other Mutated Splicing Genes with a Prognostic Value: U2AF2, PRPF8

#### 5.2.1. U2AF2 Gene (U2 Small Nuclear RNA Auxiliary Factor 2)

The *U2AF2* gene (U2 small nuclear RNA auxiliary factor 2), also known as *U2AF65* is located at 19q13.42 band. It is a heterodimeric partner of U2AF1 pre-mRNA splicing factor. *U2AF2* mutations mainly affect RNA recognition motifs (RRM1 and RRM2) [64]. These motifs recognize a polypyrimidine tract preceding the major class of 3′ splice sites (Figure 2). U2AF2 protein interacts with other splicing factors involved in MDS, such as SF3B complex in U2 complex [121], ZRSR2 [122] and SF1 [123]. It recruits PRP19 protein (*PRPF19* gene), which regulates MDM4-mediated p53 activation inducing cellular senescence [124].

Acquired *U2AF2* mutations are involved in gene expression dysregulation in leukemia and in solid tumors. In MDS, contrary to *U2AF1* mutations, *U2FA2* mutations are rare and recurrent events in myeloid pathologies, with a frequency of 1% or less [125]. They have a prognostic value [7,126], as they are associated with high-risk MDS and AML [127].

#### 5.2.2. PRPF8 (Pre-MRNA Processing Factor 8)

*PRPF8* is located on chromosome 17 at the locus 17p13.3. This gene encodes a U5 snRNP protein (PRP8), which is a major component of the catalytic core of the spliceosome. It specifically recognizes the 5′ splice site (5′SS) within the spliceosome complex B [128,129,130]. PRP8 facilitates the folding of the U2/U6 catalytic RNA [131]. Both in yeast and humans, Prp8 interacts with the 5′SS, the branch point, the polypyrimidine tract, and the 3′SS during splicing [128,129]. It is also found in the human C* spliceosome, concomitantly to RBM22 and SLU7 [32,46] (Figure 2).

The implication of *PRPF8* in the inherited human disease Retinitis Pigmentosa has been extensively studied since 2005 [132]. However, the first article demonstrating the role of *Prpf8* in the myeloid lineage differentiation was published in 2013 in a zebrafish model [133]. In those zebrafish mutants for *Prpf8*, accumulation of aberrantly spliced transcripts retaining both U2- and U12-type introns was observed [133].

From a cohort of 875 MDS cases, Haferlach et al. demonstrated that *PRPF8* mutations/deletions in MDS significantly affect the overall survival [7]. Somatic *PRPF8* mutations and hemizygous were reported [58], with half of the cases (del(17p) or *PRPF8* variants) that showed a poor prognosis (Table 1). Alterations of *PRPF8* increase the proliferation of CD34+ primary bone marrow cells and of a myeloid cell line, as well as resulting in defects of the second step of the RNA splicing process [58]. *PRPF8* aberrations were associated with increased myeloblasts and ring sideroblasts in the absence of *SF3B1* mutations [58]. Missplicing defects were reported in primary cells from patients with *PRPF8* aberrations [58]. Of note, CD34+ cells from MDS patients with *SF3B1* mutations exhibit differential expression profile of *PRPF8* [134].

## 6. Perspectives and Conclusions

MDS are a heterogeneous group of myeloid malignancies. For years, the research efforts have been driven by the intention to categorize and stratify those malignancies to group them into homogeneous entities in order to set up proper management of these diseases and decide on the therapeutic options. Successful approaches to diagnose MDS, then categorize them for the prognostic evaluation, have consisted in their genetic characterizations in association with morphological/cytological data. Those approaches have also unveiled some understanding on the molecular pathogenesis of MDS and have led to the emergence of the concept of MDS as malignancies of the RNA splicing. The most obvious examples are the identification of recurrent genetic mutations of MDS that are involved in RNA splicing. They include, for the somatic mutations, *SF3B1*, *SRSF2*, *U2AF1*, *ZRSR2*, *LUC7L2*, and, for the constitutional and somatic mutations, *DDX41*. Taken alone, each gene can play its own distinct role in the molecular pathogenesis of MDS and triggers its own specific landscape of splicing alterations. Most of these genes are included in the recently proposed molecular IPSS-M [126]. Scientific literature accumulates on that subject but more research needs to be done (review in [135]).

Haploinsufficiency of splicing genes is however less studied. Many genes implicated in RNA splicing are located in frequently deleted chromosomes, such as in del(5q) (e.g., *HNRNPA0*, *RBM27*, *RBM22*, *SLU7*) or in del(7q) (e.g., *LUC7L2*). Those genes may be mutated or not, and they can also be lost or expressed at a sub-optimal levels for their proper function. Their roles have been described and discussed in this review. Further investigations are required to decipher their exact implication in MDS pathogenesis, their diagnostic and prognostic added values.

The RNA splicing process is highly complex, with sequential steps, multiple actors, some being early-acting spliceosomal proteins (e.g., SF3B1, U2AF1, SRSF2, and ZRSR2), and other later-acting splicing factors (e.g., PRPF8, DDX41) (Figure 2). The consequences of their mutations/deletions can be distinct or convergent. Interesting data have already been brought by several groups in this direction [84], with the identification of RNA splicing signatures associated with each splicing factor mutation. For example, different authors characterized shared sets and also distinct sets of alternative spliced RNA and the common affected biological processes in the different splicing gene mutant in MDS [84,114]. Importantly, several studies also unveiled that many genes involved in splicing are, themselves, dysregulated (up or down) or misspliced, suggesting an important amplifier process of splicing defects in the MDS pathogenesis [76,77,84,95,114,134,136].

Finally, the cumulative and synergistic effect of the splicing genes on MDS pathogenesis is still not completely known (Figure 2). Obviously, some genetic defects are exclusive and other co-occurring. The synergy of the splicing factors is less studied. For example, each protein of the trio PRP8, RBM22, and SLU7 are, together, present in the C* spliceosome complex. The effect of the presence of *PRPF8* mutation associated with *RBM22* deletion (i.e., in del(5q)), associated or not with *SLU7* deletion could be investigated. Other genes involved in a component of splicing machinery, such as *SF1*, *PRPF40B*, and *SF3A1* could be explored in MDS patients.

Many authors advocate for the inclusion of sequencing some of these splicing genes to improve the diagnosis and the prognosis of MDS, so a better understanding of these cooperations, including all types of genetic defects (mutations, deletions, gains, dysregulation of gene expression, spliced variants) will be a future step for MDS management. This could lead to new diagnostic classifications for MDS and more refined prognostic scores, for the direct benefit of MDS patients.

## Figures and Tables

**Table 1:** Most important pathogenic mutations in splicing genes potentially involved in MDS.

Gene	Most Important Pathogenic Mutations	References
*DDX41*	Germline mutations: p.M1I Somatic mutations: p.R525H Possible hemizygous deletion: del(5q)	Quesada et al., 2019 [53] Polprasert et al., 2015 [54]
*HNRNPA0*	Hemizygous deletion: del(5q) Haploinsufficiency reported	Pellagatti et al., 2006 [18]
*LUC7L2*	Hemizygous deletion: del(7q) or -7 No hot spot somatic mutations: mutations along the gene ex: p.R27X, p.R71H, p.R271, p.R279X	Makishima et al., 2012 [55] Singh et al., 2013 [56] Hershberger et al., 2021 [57]
*PRPF8*	Somatic mutation: p.D1598N	Haferlach et al., 2014 [7] Kurtovic-Kozaric et al., 2015 [58]
*RBM22*	Hemizygous deletion: del(5q) No mutation described so far (not specifically searched) Haploinsufficiency reported	Pellagatti et al., 2006 [18] Boultwood et al., 2007 [14] Soubise et al., 2022 [43]
*RBM27*	Hemizygous deletion: del(5q) No mutation described so far (not specifically searched) Haploinsufficiency reported	Boultwood et al., 2007 [14]
*SF3B1*	Somatic mutations: p.K700E, p.K666N, p.R625H/C	Papaemmanuil et al., 2011 [59] Yoshida et al., 2011 [6]
*SLU7*	Possible hemizygous deletion: del(5q) No mutation described so far (not specifically searched) Possible haploinsufficiency when deleted	
*SRSF2*	Somatic mutations: p.P95H, p.P95L, p.P95R	Yoshida et al., 2011 [6] Thol et al., 2012 [60] Kim et al., 2015 [61]
*U2AF1*	Somatic mutations: p.S34F, p.S34Y, p.Q157R, p.Q157P	Yoshida et al., 2011 [6] Tefferi et al., 2017 [62] Wang et al., 2020 [63]
*U2AF2*	Somatic mutations: p.N196K, p.G301D	Maji et al., 2021 [64]
*ZRSR2*	No hot spot somatic mutations: mutations along the gene (loss-of-function)	Yoshida et al., 2011 [6] Thol et al., 2012 [60] Haferlach et al., 2014 [7]

## Data Availability

Not applicable.
